# Systems genetics analysis of body weight and energy metabolism traits in *Drosophila melanogaster*

**DOI:** 10.1186/1471-2164-11-297

**Published:** 2010-05-11

**Authors:** Patricia Jumbo-Lucioni, Julien F Ayroles, Michelle Moses Chambers, Katherine W Jordan, Jeff Leips, Trudy FC Mackay, Maria De Luca

**Affiliations:** 1 Department of Nutrition Sciences, University of Alabama at Birmingham, Birmingham, AL 35294-3360, USA; 2 Department of Genetics, North Carolina State University, Raleigh, NC 27695, USA; 3 W. M. Keck Center for Behavioral Biology, North Carolina State University, Raleigh, NC 27695, USA; 4 Department of Biological Sciences, University of Maryland Baltimore County, Baltimore, MD 21250, USA; 5 Nutrition Obesity Research Center, University of Alabama at Birmingham, Birmingham, AL 35294, USA; 6 Diabetes Research Training Center, University of Alabama at Birmingham, Birmingham, AL 35294, USA; 7 Current Address: Department of Human Genetics, Emory University, Atlanta, GA 30322, USA

## Abstract

**Background:**

Obesity and phenotypic traits associated with this condition exhibit significant heritability in natural populations of most organisms. While a number of genes and genetic pathways have been implicated to play a role in obesity associated traits, the genetic architecture that underlies the natural variation in these traits is largely unknown. Here, we used 40 wild-derived inbred lines of *Drosophila melanogaster *to quantify genetic variation in body weight, the content of three major metabolites (glycogen, triacylglycerol, and glycerol) associated with obesity, and metabolic rate in young flies. We chose these lines because they were previously screened for variation in whole-genome transcript abundance and in several adult life-history traits, including longevity, resistance to starvation stress, chill-coma recovery, mating behavior, and competitive fitness. This enabled us not only to identify candidate genes and transcriptional networks that might explain variation for energy metabolism traits, but also to investigate the genetic interrelationships among energy metabolism, behavioral, and life-history traits that have evolved in natural populations.

**Results:**

We found significant genetically based variation in all traits. Using a genome-wide association screen for single feature polymorphisms and quantitative trait transcripts, we identified 337, 211, 237, 553, and 152 novel candidate genes associated with body weight, glycogen content, triacylglycerol storage, glycerol levels, and metabolic rate, respectively. Weighted gene co-expression analyses grouped transcripts associated with each trait in significant modules of co-expressed genes and we interpreted these modules in terms of their gene enrichment based on Gene Ontology analysis. Comparison of gene co-expression modules for traits in this study with previously determined modules for life-history traits identified significant modular pleiotropy between glycogen content, body weight, competitive fitness, and starvation resistance.

**Conclusions:**

Combining a large phenotypic dataset with information on variation in genome wide transcriptional profiles has provided insight into the complex genetic architecture underlying natural variation in traits that have been associated with obesity. Our findings suggest that understanding the maintenance of genetic variation in metabolic traits in natural populations may require that we understand more fully the degree to which these traits are genetically correlated with other traits, especially those directly affecting fitness.

## Background

Obesity is a condition characterized by an excess of adipose tissue that adversely affects human health [1]. The clinical problem of excessive adipose tissue resides in its strong association with a number of chronic diseases, such as insulin resistance, type 2 diabetes mellitus (T2DM), coronary artery disease and stroke [1]. In 2003-2004, 32.2% of the adults in the United States were obese [2]. This estimate represents a significant increase in obesity prevalence over the past 20 years, and similar trends are being observed worldwide [3]. As the rise in the incidence of obesity and related health problems continues, there is a considerable need to gain a better understanding of the etiology of obesity.

In humans, large twin, adoption, and family studies have firmly established that obesity-related traits, such as body mass index and measures of body composition (e.g., fat mass, lean mass, and percentage fat mass), are highly heritable [4-6]. Segregating variation in obesity-related traits has also been observed in natural populations of most other organisms, including invertebrates [7-9]. The general conclusion from these studies is that the genetic architecture of these traits is complex and affected by many loci, with numerous gene-by-gene interactions (e.g. epistasis) and extensive genotype-by-environment interactions reported in a diverse group of organisms [10-13]. In light of this complexity a growing body of research in humans and animal models has begun to take a more systems genetic approach with focus on identifying genetic networks that control body composition and energy metabolism traits [14-16]. Preliminary findings of such studies suggest an intricate interplay between body weight control, stress, and immune response [14,15]. They also highlight a need for studying energy metabolism traits within a broader organismal context, integrating information on variation in traits influencing energy metabolism with information on variation in life history and other energetically demanding traits. This knowledge could help to explain the origin of trade-offs among these traits in natural populations. Organisms partition dietary resources acquired from the environment among the competing demands of growth, development, reproduction, maintenance and storage [17]. Since these resources are limited, the way in which they are acquired and partitioned is critical to the fitness of the individual and often result in trade-offs between energetically demanding physiological functions [17]. There is extensive empirical data on biochemical and physiological correlates of life-history variation and trade-offs within species [18,19]. Yet little is known about genes and genetic networks responsible for generating correlations between energy metabolism and life history traits in natural populations [19]. Such knowledge is not only important for understanding many central issues in life-history evolution [19], but could also elucidate the genetic basis of natural variation in human obesity.

In the present study we quantified genetic variation in wet body weight (BW), the content of three metabolites [glycogen (GLY), triacylglycerol (TAG), and glycerol (GLYC)], and metabolic rate (MR) in 40 wild-derived lines of *D. melanogaster*. We chose *D. melanogaster *as a model system because many of the genetic mechanisms controlling lipid metabolism and energy homeostasis are evolutionarily conserved between invertebrates and mammals (reviewed in [20-22]). Thus, insights about the genetics of body weight and energy metabolism gained from *Drosophila *may also apply to mammals. Additionally, *D. melanogaster *has long been a model for understanding the genetic basis of life history variation [17,23-25]. The *Drosophila *lines used in this study were previously evaluated for several ecologically relevant traits, including longevity, resistance to starvation stress, chill-coma recovery, mating behavior, and competitive fitness, as well as for transcript abundance [26]. This provided us with the opportunity to gain invaluable insights into the molecular mechanisms underlying the interrelationships among energy metabolism, behavioral, and life-history traits that have evolved in natural populations.

## Results and Discussion

### Natural variation in body weight and energy metabolism traits

**Figure 1:**
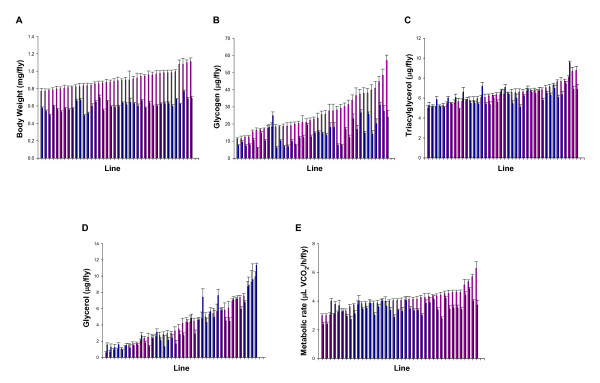
**Variation in body weight and energy metabolism traits in *D. melanogaster***. Distribution of trait means among 40 wild-derived inbred lines of *D. melanogaster*. Data represent means ± SEM for *n *= 10 independent replicates. The pink and blue bars in panels A-E depict females and males, respectively.

We found significant genetic variation among the lines for all traits analyzed (Figure 1A-F and Additional file 1: Quantitative genetic analyses of body weight and energy metabolism traits), with broad-sense heritabilities, *H^2^*, ranging from 25% to 65% in the combined sex analyses. These estimates are comparable to those found by several studies in humans [4,5,27] as well as various reports on mammalian [28,29] and non-mammalian models [7,10,30]. We also found that all traits exhibited significant sex-by-line interactions (Additional file 1: Quantitative genetic analyses of body weight and energy metabolism traits). These results, however, are most likely caused by differences in one sex in one line for some of the traits. Indeed, the genetic correlation coefficients across sexes among lines, *r_MF _*(± SEM), were very high for BW (0.94 ± 0.05; *P *< 0.0001), TAG (0.72 ± 0.11; *P *< 0.0001), and GLYC (0.97 ± 0.04; *P *< 0.0001) indicating that the same loci affect these traits in the two sexes. In contrast, moderate cross sex correlations were observed for GLY (0.44 ± 0.14; *P *= 0.0032) and MR (0.39 ± 0.15; *P *= 0.0116), suggesting that some of the variation in these traits is due to loci with sex-specific effects.

### Candidate genes for body weight and energy metabolism traits

Previously, we assessed variation in gene expression profiles among these wild-derived lines of *D. melanogaster *and identified 3,136 probes containing single feature polymorphisms (SFPs) and a total of 10,096 quantitative trait transcripts (QTTs) [26]. To identify candidate genes that might regulate variability in each of the traits quantified in this study, we performed a genome-wide association screen to search for significant associations between SFPs or QTTs with each trait [31]. At a *P*-value of 0.01, we found 93, 98, 131, 213, and 71 SFPs (see Additional file 2A: List of SFPs significantly correlated with body weight and energy metabolism traits) associated with BW, GLY, TAG, GLYC, and MR, respectively, in the analysis averaged across sexes. Because some genes were represented by 2 or more different SFPs, this analysis identified 65 independent genes for BW, 81 for GLY, 115 for TAG, 176 for GLYC, and 61 for MR. Given 3,136 SFPs, only 31 significant associations would be expected by chance at a *P *value of 0.01, thus the number of SFPs associated with each trait exceeded that expected by chance.

Based on the results of the quantitative genetic analyses described above, we also tested for association between SFPs and each trait using the data stratified by sex. The stratified analysis detected a reduced number of significant associations at a *P*-value of 0.01 (see Additional file 2B: List of SFPs significantly correlated with body weight and energy metabolism traits). The majority of these associations overlapped those identified by the analysis that used the average trait values across sexes for BW, GLYC, and TAG. However, several SFPs with sex-specific effects were detected for GLY and MR and this agrees with the moderate cross-sex correlations we observed for these traits reported above.

Our regression analyses identified 275, 130, 125, 389, and 93 QTTs significantly associated with variation in BW, GLY, TAG, GLYC, and MR, respectively, at a *P *value of 0.01 (see Additional file 3: List of transcripts significantly correlated with body weight and energy metabolism traits). In this case the number of transcripts associated with MR did not exceed chance expectation (100 significant associations would be expected); however the QTTs grouped into biologically meaningful modules as detailed below. Only few QTTs were also found as candidate genes by the SFP analyses.

**Figure 2:**
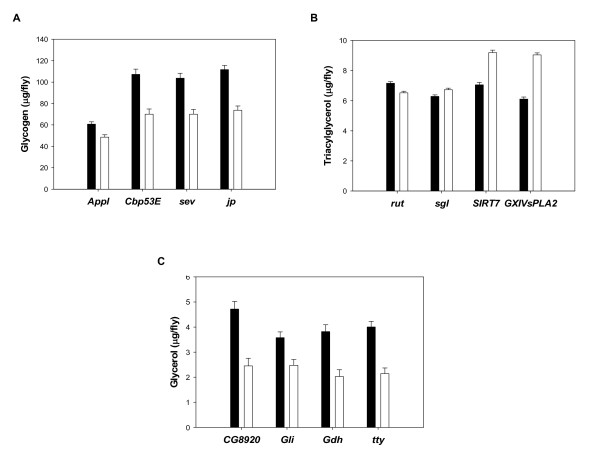
**Metabolites with significantly different levels in *P[GT1] *and *PiggyBac *transposon insertional mutations as compared to control strains**. Data represent least square means ± SEM of GLY (panel A), TAG (panel B), and GLYC (panel C) calculated using total protein content as a covariate in the analysis averaged across sexes (*n *= 20 independent replicates). Black and white bars represent mutant and control flies, respectively.

To independently validate the finding that some of the genes identified by our analysis affect the traits, we focused on candidates associated with variation in GLY, TAG, and GLYC for which homozygous *P*-element and *PiggyBac *mutations have been generated in an isogenic background. This approach has been highly effective in validating candidate genes affecting complex traits that were previously identified by expression profiling [26,32-34]. We selected five candidate genes for GLY: *β amyloid protein precursor-like *(*Appl*), *Calbindin 53E (Cbp53E), transferrin 1 *(*Tsf1*), *sevenless *(*sev*), and *junctophilin *(*jp*). We then tested for phenotypic differences between homozygous mutants of these genes and their controls. After Bonferroni correction for multiple tests, we found that four of the mutant alleles showed a significant difference in GLY compared to the control (see Additional file 4: Results of the screen of *P*-element insert lines for alterations in energy metabolites). Flies with mutations in all four genes have more GLY than the control strain (Figure 2A). *Appl *encodes an amyloid precursor-like protein that is involved in axonal transport and neuronal viability [35]. *Cbp53E *encodes a calcium-binding protein that modulates the activation of many intracellular effector proteins [35]. *Sev *encodes a tyrosine kinase receptor required for photoreceptor fate specification in the developing eye [35]. Notably, components of the *sev *signaling pathway have been previously linked to the regulation of glucose and lipid homeostasis via insulin signaling [36]. Finally, *jp *encodes a protein belonging to a novel group of highly conserved transmembrane proteins mediating optimal ionic signaling among excitable cells [35].

We selected six candidate genes for TAG: *rutabaga *(*rut*), *dead-box-1 *(*Ddx1*), *sugarless *(*sgl*), *Sirt7*, *RhoGAP71E*, and *GXIVsPLA*. We found that four of the mutant alleles showed a significant difference in TAG compared to the control (see Additional file 4: Results of the screen of *P*-element insert lines for alterations in energy metabolites). While flies with a mutation in *rut *have more TAG than the control strain, flies with mutations in *sgl*, *Sirt7*, and *GXIVsPLA *have all less TAG than the controls (Figure 2B). *rut *encodes a Ca^2+^/calmodulin-responsive adenylyl cyclase that is involved in learning and memory [35] and also has a role in food choice behavior [37]. *Sgl *encodes a homolog of mammalian UDP-glucose dehydrogenase, which is implicated in proteoglycan synthesis [35]. *P*-element insertions within the *sgl *coding region have been previously reported to significantly impact fly energy stores [38]. *Sirt7 *is a member of the Sirtuins or Sir2 (silent information regulator 2) histone deacetylase enzyme family, which has been shown to play a role in energy homeostasis and lifespan [39]. Finally, *GXIVsPLA2 *encodes an enzyme involved in phospholipid metabolism [35].

We selected six candidate genes for GLYC: *b4GalNAcTA*, *CG5946*, *CG8920*, *Gliotactin *(*gli*), *Glutamate dehydrogenase *(*Gdh*), and *tweety *(*tty*). We found that four of the mutant alleles showed a significant difference in GLYC compared to the control (see Additional file 4: Results of the screen of *P*-element insert lines for alterations in energy metabolites). Flies with mutations in all four genes have more GLYC than the control strain (Figure 2C). *CG8920 *is predicted to encode a protein belonging to the Tudor domain family [35], which binds to RNA and single-strand DNA-associated complexes in the nucleus [40]. *Gdh *encodes a nuclear-encoded mitochondrial enzyme with a role in utilization of metabolite pools for energy production [35]. *gli *encodes a transmembrane protein transiently expressed in peripheral glia whose loss of function has been implicated in defects in axonal guidance and synaptogenesis [35]. Finally, *tty *encodes a highly conserved calcium-activated chloride channel associated to flight behavioral abnormalities [35].

Together with our expression data, the findings using mutant and control stocks imply that the genes described above are candidates in the control of energy metabolites and motivate future studies to elucidate the mechanisms by which they influence metabolism.

### Transcriptional networks associated with body weight and energy metabolism traits

**Figure 3:**
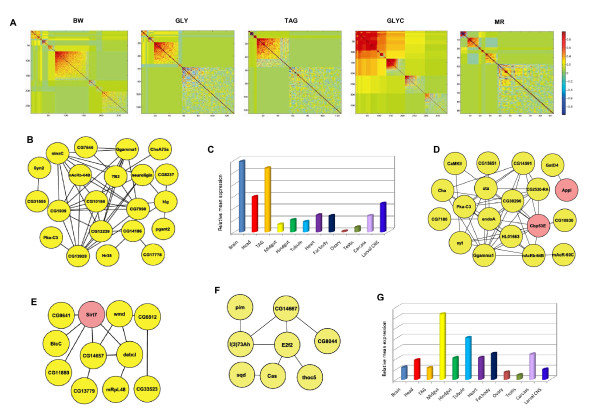
**Modules of correlated transcripts associated with variation in body weight and energy metabolism traits**. (A) Heat map of correlated probe sets after module formation for BW (13 modules), GLY (9 modules), TAG (5 modules), GLYC (13 modules), and MR (6 modules). Each point represents the correlation between two genes. The color scale bar indicates the value of the correlation. (B) Interaction network of correlated (|r| ≥ 0.7) transcripts for BW module 10. Each node represents a gene and each edge a significant correlation between a pair of genes. (C) Distribution of tissue-specific expression of transcripts in BW module 10 based on data from FlyAtlas http://www.flyatlas.org/[49]. (D) Interaction network of correlated (|r| ≥ 0.6) transcripts for GLY module 7. (E) Network of correlated (|r| ≥ 0.7) transcripts for TAG module 4. (F) Interaction network of correlated (|r| ≥ 0.9) transcripts for GLYC module 11. (G) Distribution of tissue-specific expression of all transcripts associated with MR. Nodes showed as pink in the interaction networks represent those candidate genes for which homozygous mutants were tested. The visualization of interaction networks was performed using Cytoscape 2.6.3. [84].

To provide insight into how variation in the QTTs can give rise to variation in the associated traits, we used a weighted gene co-expression network analysis [26]. Based on the fact that the transcriptome exhibits high rates of correlation between transcripts [26], this analysis groups the QTTs associated with each trait into clusters (modules) of genetically correlated transcripts. The results of the analysis are viewed in Figure 3A as a network heat map plot (interconnectivity plot) of correlated probe sets after module formation. The correlated transcript modules can also be represented as an interaction network, with edges between transcripts in the network determined by genetic correlations in transcript abundance exceeding a threshold value. This structure allows one to visualize the most highly connected genes or intramodular hub genes, which become immediate candidates for future studies. Examples of such interaction networks are reported in Figure 3 and described below.

#### Body weight

We identified 13 modules of correlated transcripts associated with BW, ranging from 2 to 97 probe sets (see Additional file 5: Modules of correlated transcripts associated with body weight and energy metabolism traits). We used the EASE (Expression Analysis Systematic Explorer) analysis implemented in DAVID functional annotation tool http://david.niaid.nih.gov/david/ease.htm[41,42] to analyze the candidate genes contained within each module for functional enrichment. Additional file 6 (see Additional file 6: Over-representation of Gene Ontology Categories, KEGG Pathways and Keywords for transcripts associated with body weight and energy metabolism traits) reports significant Gene Ontology categories. Two interesting patterns emerged from our analysis. First, after correction for multiple comparisons, we found that module 1 (*P *= 2.2E-2), module 2 (*P *= 2.3E-7), and module 13 (*P *= 1.2E-4) were enriched for genes involved in defense response categories. These results add to previous findings from systems genetics studies in mice showing that genes involved in immune function are involved in body weight regulation [15]. Evidence for this link is also found in the evolutionarily conserved role that the Toll signaling pathway plays in mediating the insulin/insulin-like growth factor (IGF) signaling. Activation of the Toll-like receptors by adipose-derived inflammatory signals, such as free fatty acids and tumor necrosis factor-α, is critical in the development of systemic insulin resistance in obese rodents and humans [43]. Recently, Diangelo *et al*. [44] reported that the activation of the immune Toll signaling pathway selectively in the fat body of *D. melanogaster *also suppresses insulin/IGF signaling leading to a decrease in both nutrient stores and growth. A major difference between flies and mammals is that while the effects on the insulin signaling in the latter are mediated by the c-Jun NH2 terminal kinase (JNK) branch of the mitogen-activated protein (MAPK) kinase signaling [43], this appears to not be the case in flies [44]. Yet, JNK is a potent antagonist of insulin/IGF signaling in both *Drosophila *and mammals [45] and is required for the immune response to gram negative bacteria in *Drosophila *[46].

The second pattern observed in our analysis is that module 10 was enriched in genes involved in cell communication (*P *= 6.9E-7) and signal transduction (*P *= 2.5E-6) and two of its hub genes, *g protein γ 1 *(*Ggamma1*) and *klingon *(*klg*) (Figure 3B), are known to be involved in neuronal development [47,48]. Tissue-specific expression patterns, based on data from FlyAtlas [49], showed that transcripts in this module are enriched in the adult brain, head, and the thoracicoabdominal ganglion, as well as in the larval central nervous system (CNS) (Figure 3C). Collectively, these findings suggest that the CNS plays a major part in regulating variation in BW in *D. melanogaster*. This is consistent with earlier reports on the genetic basis of rare monogenic forms of obesity in humans [50] and single-gene approaches in mammalian models [51,52], which have long suggested involvement of the CNS in the regulation of mammalian body weight [53]. Furthermore, recent genome-wide association studies (GWAS) in humans have mapped body weight-associated loci near genes that are highly expressed in the brain, particularly in the hypothalamus, and are involved in neuronal development and activity [54]. Our results also corroborate emerging evidence showing that, as in mammals, the CNS of *Drosophila *integrates information regarding nutrient status and stores with visual, olfactory, and taste stimuli to elicit appropriate feeding behavior responses [22,55-57]. For example, the hugin neuronal circuit modulates feeding behavior by interconnecting the gustatory sensillae to the protocerebrum, the ventral nerve cord, the ring gland, and the pharynx via the subesophageal ganglion [56]. Consistent with this finding, we identified the *hugin *(*hug*) gene as a candidate regulating variation in BW among the *Drosophila *Raleigh lines. We also found that *hug *transcript abundance is highly correlated with *Activating transcription factor-2 *(*Atf-2*), which encodes a member of the ATF/cAMP response element-binding protein family of transcription factors and has been shown to regulate fat metabolism in the fat body [58], the fly equivalent of mammalian adipose/liver tissue.

#### Glycogen

We identified 9 modules of correlated transcripts associated with GLY, ranging from 2 to 80 probe sets (see Additional file 5: Modules of correlated transcripts associated with body weight and energy metabolism traits). We found that modules 2 and 4 were enriched for genes involved in photoreceptor activity (*P *= 1.1E-3), and phospholipase A1 activity (*P *= 1.4E-3), respectively (see Additional file 6: Over-representation of Gene Ontology Categories, KEGG Pathways and Keywords for transcripts associated with body weight and energy metabolism traits).

Module 7 was enriched in genes mediating transmission of nerve impulse (*P *= 2.0E-4) and one of its major hub genes is *Cbp53E *(Figure 3D). Notably, *Cbp53E *had the highest correlation with *CG10830*, which is predicted to encode a homolog of the human *potassium channel tetramerisation domain containing 12 *(*KCTD12*) gene [35]. A recent GWAS showed that a single nucleotide polymorphism in the human *KCTD12 *gene was associated with T2DM in a French population [59]. Taken together with our findings, this proposes *KCTD12 *as a strong candidate for T2DM in humans.

Finally, it is worth mentioning that two of the genes in GLY module 6, *puckered *(*puc*) and *hemipterous *(*hep*), encode a MAP kinase phosphatase and a MAP kinase, respectively, that regulate the JNK signaling pathway [35]. The mechanism by which changes in these genes regulate variation in GLY levels is not known, however, previous studies have shown that JNK represses *Drosophila *insulin-like peptide transcription in the neurosecretory cells of the brain that produce them [60]. Thus, a plausible mechanism is that variation in *puc *and *hep *modulates GLY by regulating insulin-like peptide secretion via the JNK pathway. This hypothesis however needs to be tested in future studies.

#### Triacylglycerol

We identified 5 modules of correlated transcripts associated with TAG, ranging from 1 to 71 probe sets (see Additional file 5: Modules of correlated transcripts associated with body weight and energy metabolism traits). Notably, seven of the genes in TAG module 4, *death executioner Bcl-2 homologue *(*debcl*), *sex combs extra *(*Sce*), *viral iap-associated factor *(*viaf*), *Sirt7*, *CG7516, GXIVsPLA2*, and *Srp54*, have human homologs, *BOK*, *RING1*, *Pdcl3*, *SIRT7*, *NOL10*, *PLA2G12A*, and *SFRS12*, respectively, whose transcript abundance has been associated with obesity in mice using a systems genetics analysis [15]. Visualization of module 4 illustrates that two of these genes, *Sirt7 *and *debcl*, are hubs (Figure 3E). As discussed above, *Sirt7 *is involved in chromatin silencing and its mouse ortholog has been recently reported to play a role in the regulation of stress response of cardiomyocytes and to prevent apoptosis and inflammatory cardiomyopathy [61]. *debcl *is a member of the evolutionarily conserved Bcl-2 family of protooncogenes that is composed of both pro- (*e. g*. bax and bak) and anti-apoptotic (*e. g*. bcl-2 and bcl-xL) proteins [62]. Collectively, these data argue that variation in genes involved in apoptosis control TAG accumulation. As fatty acids destined for oxidation are in part derived from stored TAGs, our data are consistent with studies in mammals reporting that oxidation of fatty acids is inhibited by several mitogenic stimuli and increased by various agents of growth arrest and/or apoptosis [63]. Our results not only suggest that the link between TAG accumulation and apoptosis is evolutionarily conserved, but also indicate that it extends to the transcriptional level.

#### Glycerol

We identified 13 modules associated with GLYC, ranging from 2 to 101 probe sets (see Additional file 5: Analysis of modules of correlated transcripts associated with body weight and energy metabolism traits). None of the modules associated with GLYC were found to be significantly enriched in specific functional categories after Benjamini correction. However, two points are worthy of mention. First, the most highly connected gene in module 11 was *E2F transcription factor 2 *(*E2f2*) (Figure 3F) that is critical for cell-cycle arrest [64]. Since glycerol is an important intermediate in TAG metabolism, these latter findings corroborate the hypothesis of a link between cellular progression, apoptosis, and fatty acids oxidation discussed above. Second, among the genes included in module 12, there is *Glycerol 3 phosphate dehydrogenase *(*Gpdh*) that plays a major role in the metabolism of carbohydrates for insect flight [65].

#### Metabolic rate

We identified 6 modules of correlated transcripts associated with MR, ranging from 3 to 58 probe sets (see Additional file 5: Modules of correlated transcripts associated with body weight and energy metabolism traits). We found that transcripts associated with variation in MR were enriched for genes involved in hydrolase (module 2, *P *= 2.6E-3) and alpha-glucosidase (module 5, *P *= 6.8E-05) activities (see Additional file 6: Over-representation of Gene Ontology Categories, KEGG Pathways and Keywords for transcripts associated with body weight and energy metabolism traits). Transcripts associated with MR were also enriched for genes that are mainly expressed in the midgut and the Malpighian tubules (Figure 3G). The Malphighian tubules in insects are part of the excretory system responsible for absorbing water and nitrogenous wastes from the haemolymph and so critical for maintaining proper internal osmotic conditions. Because of their small size, insects and other terrestrial arthropods are susceptible to water loss by evaporation through the cuticle. One process that also promotes water loss in insects and thus increases the danger of desiccation is the respiratory gas exchange [66]. Reduction in metabolic rate and the demand for oxygen has been proposed as a mechanism that can help the fly to conserve water [67]. In addition, insect renal tubules constitute a cell-autonomous immune system that protects the organism against bacterial infection [68] and detoxification of xenobiotics [69]. Thus, variation in gene expression in the gut and tubules could influence metabolic rate in a number of ways, through alterations in osmotic balance to changes in digestive efficiency. It will be important for future functional genetics studies to verify the phenotypic effects of variation in gene regulatory networks in these organs to elucidate their contribution to determining whole-body metabolic rates.

### Genetic correlations between energy metabolism and life-history traits

Next, we asked whether there were significant genetic correlations between the BW and the metabolic traits. While we did not find any significant correlation using all data pooled across sexes, when we analyzed the data stratified by sex we observed a correlation significant at *P *< 0.05 between MR and TAG (*r_G _*= 0.45, *P *= 0.004) in females. Additionally, we observed correlations between BW and GLY (*r_G _*= 0.32, *P *= 0.042) and BW and MR (*r_G _*= 0.38, *P *= 0.014) in males. These correlations however are not significant after correction for multiple tests based on sequential Bonferroni [70]. Although the relationships among BW and energy metabolism traits in this study are fairly weak, similar relationships have been found in other studies using *Drosophila*. For example, several laboratory selection studies in *D. melanogaster *have shown that both female and male adult flies selected for resistance to desiccation and starvation are significantly heavier and have higher GLY than unselected controls [71]. Based on these observations, we speculate that the genetic correlation between BW and GLY identified in our study may reflect the influence of these traits on the fly's ability to tolerate abiotic stresses, such as desiccation and starvation, in the wild. No data is currently available for desiccation resistance in these wild-derived flies, however, the lines were previously assessed for starvation resistance as well as other life-history traits, including competitive fitness, chill-coma recovery, copulation latency, and longevity [26]. Thus, we sought to test for genetic correlations between these life-history traits and the traits measured in this study. The results of the analysis are shown in Table 1. In accord with the selection studies discussed above, we found a significant positive correlation between BW and starvation resistance in both male and female flies (Table 1). Consistently, a weak correlation was also observed between GLY and starvation resistance in both sexes. Interestingly, we did not find any correlation between TAG and starvation resistance (Table 1). This is in contrast to selection studies that have long suggested that an increase in lipid stores may be an important mechanism underlying evolution of greater starvation resistance [72]. A possible explanation for this result is that the relationship between fat reserves and starvation may be a consequence of laboratory selection. This idea is supported by the fact that previous studies performed by Hoffmann *et al*. [73], who used isofemale lines derived from wild populations, also did not observe any correlation between lipid storage and starvation resistance. Finally, we showed negative correlations between BW or GLY and competitive fitness (Table 1), suggesting that the ability to access glycogen resources may be a mechanism responsible for the life history trade-off between growth and fitness.

**Table 1:** Genetic correlations between energy metabolism and life-history traits averaged across sexes (A), for females (B), and for males (C).

	FT	CL	SR	CC	LS
**A**					
BW	-0.48 ± 0.14**	0.21 ± 0.16	0.52 ± 0.14***	0.32 ± 0.15	0.01 ± 0.16
GLY	-0.38 ± 0.15**	0.08 ± 0.16	0.29 ± 0.15	0.02 ± 0.16	0.17 ± 0.16
TAG	-0.14 ± 0.16	0.08 ± 0.16	0.12 ± 0.16	0.18 ± 0.16	-0.15 ± 0.16
GLYC	0.05 ± 0.16	0.22 ± 0.16	0.06 ± 0.16	0.01 ± 0.16	-0.07 ± 0.16
MR	0.14 ± 0.16	-0.11 ± 0.16	-0.04 ± 0.16	0.01 ± 0.16	-0.26 ± 0.16
					
**B**					
BW	-0.52 ± 0.14***	0.38 ± 0.15*	0.53 ± 0.14***	0.26 ± 0.16	-0.01 ± 0.16
GLY	-0.27 ± 0.16	0.10 ± 0.16	0.36 ± 0.15*	-0.07 ± 0.16	0.10 ± 0.16
TAG	-0.10 ± 0.16	0.20 ± 0.16	0.14 ± 0.16	0.05 ± 0.16	0.01 ± 0.16
GLYC	0.04 ± 0.16	0.22 ± 0.16	0.00 ± 0.16	0.11 ± 0.16	-0.08 ± 0.16
MR	0.06 ± 0.16	-0.11 ± 0.16	0.00 ± 0.16	-0.10 ± 0.16	-0.16 ± 0.16
					
**C**					
BW	-0.42 ± 0.15**	-0.06 ± 0.16	0.67 ± 0.12****	0.27 ± 0.16	0.07 ± 0.16
GLY	-0.59 ± 0.13****	0.04 ± 0.16	0.42 ± 0.15**	0.20 ± 0.16	0.24 ± 0.16
TAG	-0.17 ± 0.16	-0.04 ± 0.16	0.14 ± 0.16	0.38 ± 0.15**	-0.29 ± 0.16
GLYC	0.06 ± 0.16	0.22 ± 0.16	0.13 ± 0.16	-0.05 ± 0.16	-0.06 ± 0.16
MR	0.25 ± 0.16	-0.10 ± 0.16	-0.04 ± 0.16	-0.15 ± 0.16	-0.41 ± 0.15**

BW: body weight; GLY: glycogen; TAG: triacylglycerol; GLYC: glycerol; MR: metabolic rate; FT: competitive fitness; CL: copulation latency; SR: starvation resistance; CC: chill-coma recovery; LS: lifespan. *P ≤ 0.05; **P ≤ 0.01; ***P ≤ 0.001; ****P < 0.0001. Values in bold indicate correlations that remain significant after Bonferroni correction based on sequential Bonferroni tests.

To gain insight into the molecular basis of the observed genetic correlations, we tested whether there was significant overlap of common transcripts between modules for the energy metabolism traits and life-history traits. We found substantial modular pleiotropy between BW, competitive fitness, and starvation resistance (see Additional file 7: Modular pleiotropy between energy metabolism and life history traits). In particular, we observed that transcript abundance of genes involved in innate immune response, such as *Attacin-C *(*AttC*), *Cecropin C *(*CecC*), and *PGRP-SB1*, were associated with variation in all three traits. Large energy investments are necessary for an adequate immune system to fight infections [74]. As life-history theory predicts that the amount of energy available is finite [17], maintaining the cellular and molecular capabilities of mounting an efficient immune response may not be possible without cost to other energy demanding physiological functions. Indeed, trade-offs between immune function and other traits involving competition, specifically larval competitive ability in *Drosophila*, have been extensively reported [75,76]. Based on these observations, we speculate that genotypic differences in the efficiency of the immune response among wild-derived lines of *D. melanogaster *may reflect differences in allocation of resources between traits associated with survival in a way that maximizes fitness. This view is consistent with evidence of an evolutionarily conserved link between immune function and the insulin/IGF signaling discussed above. These *Drosophila *lines are currently being assessed for variation in the efficacy of their immune response to infection which will allow us to test this hypothesis.

Our analysis also showed significant modular pleiotropy between BW, GLY, and competitive fitness (see Additional file 7: Modular pleiotropy between energy metabolism and life history traits). Three genes, *nicotinic Acetylcholine Receptor beta 64B *(*nAcRβ-64B*), *Diuretic hormone 31 receptor 1 *(*Dh31-R1*), and *cAMP-dependent protein kinase 3 *(*Pka-C3*) were associated with variation in all the traits. *nAcRβ-64B *and *Dh31-R1 *encode predicted G-protein coupled receptors that bind to the neurotransmitter acetylcholine and a diuretic hormone, respectively [35]. *Pka-C3 *encodes a cAMP-dependent protein kinase [35] whose transcription is regulated by light [77]. Furthermore, we found that two photosensory opsins, *rhodopsin 4 *(*Rh4*) and *rhodopsin 6 *(*Rh6*), were associated with variation in GLY and competitive fitness. In insects, the transcriptional coordination of circadian clocks has been implicated in affecting life-history traits by regulating physiological and behavioral rhythms [77], including feeding rhythms [78]. Daily light/dark cycles may affect circadian rhythms by either entraining the clocks or via clock-independent molecular pathways and *Drosophila *circadian photoreception is mediated by cryptochrome in clock neurons and by rhodopsins in photic organs [79]. Based on these observations, our findings confirm the interrelations among circadian photoreception, life-history traits, and energy metabolism and identify a key set of transcripts involved in this process.

## Conclusions

The present study identified a large number of genes that varied both at the level of DNA sequence and at the level of gene expression to produce natural variation in BW, the content of GLY, TAG and GLYC, and MR among 40 wild-derived lines of *D. melanogaster*. Candidate genes identified based on sequence polymorphism generally differed from those identified based on variation in gene expression among lines. This suggests that phenotypic variation is the product of both alterations in gene expression as well as allelic variation at the sequence level. The relative importance of these two processes in producing phenotypic variation remains to be determined, but may vary depending on the trait and sampled population. Our gene expression data identified a number of modules of co-expressed genes affecting these traits with surprisingly little overlap. As these modules contain many genes of unknown function, their co-occurrence with genes with known function related to specific traits may be useful for annotation purposes. We did identify significant modular pleiotropy between BW, GLY, and competitive fitness and future studies will need to explore and validate the functional genetic basis of these interrelationships. Such knowledge would be useful not only in a practical sense to predict correlated changes in related traits given medical interventions to control body weight, but also from an evolutionary standpoint to elucidate the extent to which such pleiotropic modules might guide and constrain the evolution of the affected traits.

## Methods

### *Drosophila *stocks

The 40 unrelated wild-type inbred lines of *D. melanogaster *were established from a sample of isofemale lines collected in the Raleigh Farmer's market (NC) and inbred to near-homozygosity by 20 generations of full-sib inbreeding [26]. Mutants and their co-isogenic control lines were obtained from the Bloomington *Drosophila *Stock Center http://www.flybase.org.

We maintained each stock at constant parental density for at least two generations to minimize environmental effects. To control for larval density, we allowed the parents of the experimental flies to mate for 3 hours to generate egg collections on apple juice/agar medium in laying plates. After 24 hours, we picked groups of 100 first-instar larvae from the surface of the medium and put into replicate vials. For all assays, we used ten replicate vials per line, with each vial containing a group of 10 single-sexed individuals aged 3-5 days. We reared flies under the same experimental conditions described in Ayroles *et al*. [26], *i.e*. standard cornmeal, agar, molasses, and yeast medium, 25°C, 60-75% humidity, and 12 hr/12 hr light/dark cycle.

### Body weight and metabolite measurements

We first starved the flies for one hour under non-dehydrating conditions to reduce the food-derived TAG and GLY present in the gut [80]. We then weighed each group of flies to 0.1 mg accuracy with an analytical balance and stored them at -70°C. Finally, we homogenized each group using the protocol described in [7,81] and measured TAG and GLYC spectrophotometrically using a commercially available kit (Sigma-Triglyceride Assay Kit) following the manufacturer's suggested protocol.

GLY was measured from the same homogenates using a modification of the protocol described in Clark *et al*. [81]. Briefly, aliquots of 1.67 μl of homogenate were added to 250 μl of a reagent containing 0.1 U/ml of amyloglucosidase, 5 U/ml of glucose oxidase, 1 U/ml of peroxidase, and 0.04 mg/ml of O-dianisidine dihydrochloride. After 30-minute incubation period at 37°C, OD_540 _was measured. Concentration of GLY was determined from glucose and glycogen standards run with each replicate. Each sample was assayed twice and the mean used in the analysis. Previous studies have shown that this protocol accurately reflects glycogen concentration and that endogenous glucose present in the flies contributes only negligibly to the results [81].

### Metabolic rate measurements

We measured MR as CO_2 _production using a flow-through respirometry system (Qubit System Research, Kingston, Ontario, Canada) and a modification of the method described in Van Voorhies *et al*. [82]. Briefly, a pump is used to push air through a CO_2 _scrubber therefore providing CO_2_-free air to the system. The airstream is saturated with H_2_O by passing through a series of gas syringes filled with sterile H_2_O and cotton wool. Pressure in the line is controlled by a precision pressure regulator that sets the input pressure to the 4-channel mass flow meter/controller where the flow is divided into 4 gas streams and provided to the sample chambers. The flow rate entering the chamber was 30 ml/min. After leaving the sample chambers, air enriched in CO_2 _enters into the 4-channel gas switcher that directs the flow to either the analysis system or to waste (vented). For the determination of CO_2_, sample air was pulled through a drying column to remove H_2_O, a mass flow meter, and then the CO_2 _analyzer that has a range of 0-2000 ppm CO_2 _with a resolution of better than 1 ppm. We measured CO_2 _for 10 minutes/chamber with a 30 second flush period between measurements. The amount of CO_2 _produced by each group of flies was calculated using C950 Data Acquisition software (Qubit System Research, Kingston, Ontario, Canada).

### Quantitative genetic analyses

All statistical analyses were performed using SAS version 9.1. We used a mixed model ANOVA to partition variation in each trait among the inbred lines according to the model, *Y *= *μ *+ *L *+ *S *+ *L *× *S *+ *E*, where *μ *is the overall mean; *L *and *S *are the main effects of Line (Random) and Sex (Fixed); *LxS *is the random effect of sex-by-line interaction; and *E *is the within-vial error variance. Reduced models by sex were also run. Broad-sense heritabilities (*H^2^*) were computed as *H*^2 ^= (*σ_L_*^2 ^+ *σ_LS_*^2^)/(*σ_L_*^2 ^+ *σ_LS_*^2 ^+ *σ_E_*^2^) for the analyses pooled across sexes, where *σ_L_^2^, σ_LS_^2^*, and *σ_E_^2 ^*are the among line, sex-by-line and within line variance components, respectively. Cross-sex genetic correlations (*r_MF_*) were also estimated as *r_MF _= cov_♀♂_/(σ_♀_σ_♂_)*, where *cov_♀♂ _*is the covariance of line means between females and males, and *σ_♀ _*and *σ_♂ _*are the square roots of the among line variance components for females and males, respectively. Genetic correlations between traits were calculated as *r_GT _*= *cov_G12 _*/(*σ_G1_σ_G2_*), where *cov_G12 _*is the covariance between traits among line means from the joint analysis, and *σ_G1 _*and *σ_G_*_2 _are the square roots of the variances among lines from the analyses of each trait separately. We used sequential Bonferroni procedure to correct for multiple tests of significance of correlation coefficients among traits [70]. The coefficients of genetic (*CV_G_*) and environmental (*CV_E_*) variances were calculated as *CV_G _*= 100*σ_G_*/*μ *and *CV_E _*= 100*σ_E_*/*μ*, respectively, where *σ_G _*and *σ_E _*are the square roots of the line and within line variance components, respectively.

### Transcript-trait and SFP-trait associations

To identify transcripts associated (*P *< 0.01) with variation in each trait we performed a regression analysis as previously described [24]. Briefly, regression models of the form *Y *= *μ *+ *S *+ *T *+ *S *× *T *+ *ε*, where *S *is sex, *T *is the trait, and *ε *is the error term were computed for each probe set. Similarly, SFPs associated (*P *< 0.01) with each trait were identified using the ANOVA model *Y *= *μ *+ *M *+ *S *+ *S *× *M *+ *ε*, where *M *is the presence or absence of the SFP, *S *is sex, and *ε *is the error term. Reduced models by sex were also run.

### Transcriptional network

The genetic correlations between all transcripts significantly associated with each trait were computed after removing the correlation between these transcripts and the trait. This was achieved by fitting the model *Y *= *μ *+ *E *+ *S *+ *E *× *S *+ *ε *(*Y *is the trait, *E *is the covariate median log2 expression level, *S *is the sex effect and *ε *the residual error) and extracting the residuals to compute pair-wise transcript correlations for module construction [26]. Modules of transcripts associated with each trait with coordinated patterns of expression across the 40 lines were then quantified as described previously [83] by transforming the pairwise genetic correlations among transcripts into Euclidean-like distances, which were used to construct an affinity matrix. The transcripts were partitioned into modules using a graph-theoretical approach that envisions the transcripts as nodes in an undirected graph whose edges are weighted by the entries of the affinity matrix. Transcriptional modules common to a metabolic trait as well as to other traits measured on the 40 wild-derived inbred lines [26] were identified by comparing the transcripts in each metabolic module to the transcripts in each module from the other traits and comparing whether the overlap between the modules exceed what is expected by chance using a Fischer's exact test.

## Authors' contributions

MD and TFCM conceived the study and participated in its design and coordination. PJL and MMC performed research. JFA, KWJ, PJL, and JL analyzed the data. MD wrote the paper. All authors read, critically revised the manuscript, and approved the final manuscript.

## Acknowledgements

We are grateful to Dr Brett McKinney for contributing with analytic tools. We thank two anonymous reviewers for their valuable suggestions. We also thank Dr Barbara Gower for her technical assistance in the metabolite measurements. Dr Gower's laboratory is supported, in part, by NIH Grants P30-DK56336 (NORC), P60-DK079626 (DRTC), and UL1RR025777 (CCTS). This study was supported by NIH Grants R01 DK084219 to MDL and JL and R01 GM 45146 to TFCM.

## Supplementary Material

Additional file 1 **Quantitative genetic analyses of body weight and energy metabolism traits**. This file includes estimates of variance components among 40 wild-derived inbred lines of *D. melanogaster *for the combined sex analyses. Click here for file

Additional file 2 **List of SFPs significantly correlated with body weight and energy metabolism traits in the analyses averaged across sexes (panel A) and stratified by sex (panel B)**. This file includes Affymetrix Probe identification number, FlyBase accession number and name of the gene tagged by the SFP, probe containing SFP, *P-*value from the ANOVA of the difference in trait means between the two SFP classes, minor allele frequency of the SFP, trait mean for lines with the common and minor alleles, and gene ontology information. SFPs included were significant at uncorrected *P *< 0.01. Genes detected by the two analyses are highlighted in gray in panel B. Click here for file

Additional file 3 **List of transcripts significantly correlated with body weight and energy metabolism traits**. This file includes Affymetrix Probe identification number, FlyBase accession number and name of the gene transcript, *P-*value from the regression analyses, mean expression level, broad-sense heritability, and gene ontology information. Transcripts included were significant at uncorrected *P *< 0.01. Click here for file

Additional file 4 **Results of the screen of *P*-element insert lines for alterations in energy metabolites**. This file shows the genes tagged by the *P*-elements, the *P*-element insertion sites, and *P*-values from analysis of covariance (total protein content being a covariate) comparing the mutant and control data across the sexes and stratified by sex. Click here for file

Additional file 5 **Modules of correlated transcripts associated with body weight and energy metabolism traits**. This file shows the average correlation of a transcript with all other transcripts in its module (degree) and the average correlation of all transcripts in the module (average degree). Click here for file

Additional file 6 **Over-representation of Gene Ontology Categories, KEGG Pathways, and Keywords for transcripts associated with body weight and energy metabolism traits**. This file shows the results of the over-representation analysis of transcript modules performed with EASE. Included are the number of genes in the annotation category (count), the number of genes in the annotation category/total number of significant genes (%), and the *P *value from a modified Fisher exact test for enrichment of genes in an annotation category. The file reports only results that survived Benjamini correction *P*-values of 0.05 or less. Click here for file

Additional file 7 **Modular pleiotropy between energy metabolism and life history traits**. This file includes a list of modules of transcripts significantly overlapping between traits in this study and traits listed in [26], *P *value from a Fisher exact test, and pleiotropic genes. Click here for file
