# Supplementary material for: Systems genetics analysis of body weight and energy metabolism traits in Drosophila melanogaster
Source: BMC Genomics. 2010 May 11;11:297. doi: 10.1186/1471-2164-11-297 (PMC2880307; doi:10.1186/1471-2164-11-297)
Supplement: Additional file 1 — Quantitative genetic analyses of body weight and energy metabolism traits. This file includes estimates of variance components among 40 wild-derived inbred lines of D. melanogaster for the combined sex analyses. [file 1471-2164-11-297-S1.DOC]

**Additional file 1 - Quantitative genetics analyses of body weight and energy metabolism traits for 40 wild-derived inbred lines of *D. melanogaster***.

| Trait^a^ | Mean  (±SE) | *σ_L_*^2b^ | | *σ_SL_*^2c^ | | *σ_G_*^2d^ | | *σ_E_*^2e^ | | *σ_P_*^2f^ | | *H^2g^* | | CV_G_^h^ | | CV_E_^i^ | |
| --- | --- | --- | --- | --- | --- | --- | --- | --- | --- | --- | --- | --- | --- | --- | --- | --- | --- |
| BW | 0.75  (± 0.01) | 0.02^**^ | | 0.01^****^ | | 0.03 | | 0.09 | | 0.12 | | 0.25 | | 23.09 | | 40.00 | |
| GLY | 6.02  (± 0.14) | 30.37^**^ | | 31.23^****^ | | 61.60 | | 43.42 | | 105.02 | | 0.59 | | 130.37 | | 109.46 | |
| TAG | 6.27  (± 0.04) | 0.42^****^ | | 0.18^****^ | | 0.60 | | 0.64 | | 1.24 | | 0.48 | | 12.35 | | 12.76 | |
| GLYC | 4.11  (± 0.11) | 0.23^****^ | | 0.01^**^ | | 0.24 | | 0.13 | | 0.37 | | 0.65 | | 11.92 | | 8.77 | |
| MR | 3.80  (± 0.03) | 0.12^*^ | 0.14^****^ | | 0.26 | | 0.36 | | 0.62 | | 0.42 | | 13.42 | | 15.79 | |  |

^a^ BW: body weight; GLY: glycogen; TAG: triacylglycerol; GLYC: glycerol; MR: metabolic rate. ^b^ Among line variance component. ^c^ Sex by line interaction variance component. ^d^ Total genetic variance (*σ_L_*^2^ *+σ_SL_*^2^). ^e^ Variance within lines. ^f^ Total phenotypic variance (*σ_G_*^2^ *+σ_E_*^2^). ***^g^*** Broad-sense heritability (*σ_G_*^2^ */σ_P_*^2^). ^h^ Coefficient of genetic variation (100*σ_G_**/*Mean). ^i^ Coefficient of environmental variation (100*σ_E_/*Mean). ^*^*P* <0.05; ^**^*P* <0.01; ^***^*P* <0.001; ^****^*P* <0.0001
